# I-COMS: Interprotein-COrrelated Mutations Server

**DOI:** 10.1093/nar/gkv572

**Published:** 2015-06-01

**Authors:** Javier Iserte, Franco L. Simonetti, Diego J. Zea, Elin Teppa, Cristina Marino-Buslje

**Affiliations:** Fundación Instituto Leloir. Av. Patricias Argentinas 435, C1405BWE, Buenos Aires, Argentina

## Abstract

Interprotein contact prediction using multiple sequence alignments (MSAs) is a useful approach to help detect protein–protein interfaces. Different computational methods have been developed in recent years as an approximation to solve this problem. However, as there are discrepancies in the results provided by them, there is still no consensus on which is the best performing methodology. To address this problem, I-COMS (interprotein COrrelated Mutations Server) is presented. I-COMS allows to estimate covariation between residues of different proteins by four different covariation methods. It provides a graphical and interactive output that helps compare results obtained using different methods. I-COMS automatically builds the required MSA for the calculation and produces a rich visualization of either intraprotein and/or interprotein covariating positions in a circos representation. Furthermore, comparison between any two methods is available as well as the overlap between any or all four methodologies. In addition, as a complementary source of information, a matrix visualization of the corresponding scores is made available and the density plot distribution of the inter, intra and inter+intra scores are calculated. Finally, all the results can be downloaded (including MSAs, scores and graphics) for comparison and visualization and/or for further analysis.

## INTRODUCTION

Recent work has demonstrated the accuracy of coevolution-based protein contact prediction using several approaches as mutual information (MI) (1–3), direct couplings (4–9) and more recently pseudo-likelihood-based approaches (8,10).

Despite all the work being done in this field, the use of residue covariation methods, using only sequence-based data for predicting protein–protein contacts, is still in an early stage of development. Interprotein coevolutionary analysis is appealing but is far from being easy. Few successful works on protein–protein interaction prediction using bacterial genomes have recently been reported (11,12). Nevertheless, setting up the analysis as well as interpreting the results is complicated and requires further analysis and discussion. The sole preparation of a suitable multiple sequence alignment (MSA) to perform the analysis is limited to highly expert users. Identifying covarying residue pairs between two proteins A and B is not a straightforward task; protein A and protein B for each organism must be properly paired in each alignment of A and B proteins. The desirable feature is to align orthologs proteins, as they are likely to perform equivalent functions, and even if they have diverged since the speciation event, they are more likely to be functional counterparts in different species than other types of homologs (12). To approximate the selection of orthologs for each protein in the presence of multiple paralogs in a single genome, Ovchinnikov *et al*. (11) have used the intergenic distance as a proxy: i.e. pair of genes with conserved chromosomal locations separated in the genome by fewer than 20 other annotated genes. Hopf *et al*. (13) apply a criterion where each concatenated protein pair must be located on the same genomic contig and must be as close as possible to each other on the genome, when compared to all other possible pairings in the same species. Nonetheless, these two approximations only apply for prokaryotic genomes and it is known that changes in function might be as common between orthologs as between paralogs (14).

Orthology assignment is a very active field in which latest achievements are summarized in (15). All-against-all sequence comparisons can be time consuming and pre-calculated databases might have confident assignments of orthologs but with a very low number of sequences as a general rule. In this work, we use the closest blast E-value as a proxy of orthology to construct a paired MSA and to retrieve a high number of sequences in a fast way. The closest hit is often interpreted to imply that the protein is the closest homolog (ortholog). Though, for genes with few homologs, the closest hit may not be the closest homolog (16). We understand that this may be a strong limitation and for this reason, we allow the user to load its own alignments based on a more accurate orthology assignment and leave this auto-assignment possibility for less expert users (or when other approach is not possible because of the low number of sequences). Here we present a server that allows users to analyze coevolutionary relationships between groups of proteins with four state of the art coevolution algorithms: precise structural contact prediction using sparse inverse covariance estimation (PSICOV) (9), Direct Coupling Analysis with mean-field approximation (mfDCA) (7,17), corrected MI (3) and the plmDCA implementation used in CCMpred, a pseudo-likelihood maximization (PLM-based) contact prediction approach (18).

Strong emphasis is given to the comparability between methods. Reported results from different methods cannot be directly compared due to differences in the used data set, i.e. they differ in the analyzed protein cases, the number of sequences in the alignments and/or the aligning method used.

Three key points are addressed in this server. First, the construction of a concatenated MSA suitable for interprotein coevolution calculation, given two or more UniProt identifiers, protein sequences or a pdb code as input. Second, we provide the user the option to run four algorithms at once obtaining the covariation between residues both intraprotein and interprotein.

Finally, we offer a visual summarization of the results, allowing a fast and comprehensive comparison between results that would help to identify the method that suits better a particular biological question.

To compute the different covariation scores across proteins, individual protein sequences must be aligned and paired up with the partner that is presumed to interact. Our approximation is to pair and concatenate proteins that have identical taxonomic ID (taxID) to ensure that both proteins belong to the same organism, a minimal condition for a putative interaction.

A visualization of either intraprotein and/or interprotein results is provided in a circos representation. Additionally comparison between any pair of methods is available as well as the overlap between all four methods. As a complementary source of information, a matrix visualization of scores is available and the density plot distribution of inter, intra and inter+intra scores is shown. Finally, all the results can be downloaded (including MSAs, scores and graphics) to further analysis, comparison and visualization.

This website is free and open to all users and there is no login requirement. URL: http://I-COMS.leloir.org.ar/.

## MATERIALS AND METHODS

### Covariation scores

Computation of the covariation scores for different methods is performed using already existing software. Corrected MI scores (from here MI for simplicity) are calculated between pairs of columns in the MSA as described in (3) using the same tools present in the MISTIC web server (19). Briefly, the frequency for each amino acid pair was calculated using sequence weighting techniques and low count corrections and was compared to the expected frequency assuming that mutations between amino acids were uncorrelated. Next, the MI was calculated as a weighted sum of the log-ratios between the observed and expected amino acid pair frequencies. The average product correction (APC) method of Dunn *et al*. (2008) (20) was applied to reduce the background MI signal for each pair of residues and the MI scores were finally translated into MI *z*-scores by comparing the MI values for each pair of position with a distribution of prediction scores obtained from a large set of randomized MSAs. The *z*-score is then calculated as the number of standard deviations that the observed MI value falls above the mean value obtained from the randomized MSAs.

mfDCA is a statistical inference framework used to infer direct coevolutionary couplings among residue pairs in MSAs. mfDCA, in opposite to original mfDCA, provides the computational power to apply this tool in a high throughput manner to a number of protein and domain families.

PSICOV introduces the use of sparse inverse covariance estimation to the problem of protein contact prediction. The method builds on work which had previously demonstrated corrections for phylogenetic and entropic correlation noise and allows accurate discrimination of direct from indirectly coupled mutation correlations in the MSA. PSICOV and mfDCA scores are computed using freecontact tool (17), which implements fast and memory-efficient algorithms for both methods.

CCMpred (downloaded from https://bitbucket.org/soedinglab/ccmpred) is a C implementation of a Markov Random Field PLM for learning protein residue–residue contacts as made popular by Ekeberg (10) and Balakrishnan (21). While predicting contacts with comparable accuracy to the referenced methods, CCMpred is written in C/CUDA C, performance-tuned and therefore much faster.

All tools compute a score for every pair of columns in a given MSA. This is computationally expensive and may take a long time to finish. This is particularly true for MSAs with a high number of columns. The number of sequences in the MSA does not affect the performance significantly.

### Paired MSA construction

To build the MSA we blast each protein queried by the user against Uniprot-KB and retrieve all sequences with an E-value threshold of 1e-5 and 3 Psi-Blast iterations. We save only one protein for each NCBI taxID. If there is more than one protein with identical taxID (i.e. paralogous proteins), the one with the lowest expected value for that query is used. In the case that the expected value is equal, the one with the higher bit-score is chosen (more likely orthologous to its query). The same methodology was used to build pairs of MSAs of orthologous proteins to calculate coevolution by Ochoa and Pazos (2010) (22). Then, to create the mutiprotein MSA, we concatenate the proteins with the same taxID (Supplementary file 1, Figure S1 and S2). To assess whether this simple procedure is acceptable for covariation calculation compared to GREMLIN procedure to construct an MSA (23), we built the paired MSAs using interprotein COrrelated Mutations Server (I-COMS) procedure for a subset of GREMLIN protein complexes. We ran mfDCA, MI and CCMpred on both the original MSAs (downloaded from GREMLIN's webpage: http://openseq.org/cplx.php?mode=pdb) and the I-COMS built MSAs. The performance of each method on both types of alignments, measured as the areas under the ROC curve for interprotein and intraprotein contact prediction as well as the extent to which the alignments are similar in terms of retrieved Uniprots and taxIDs, is provided as supplementary material (Supplementary file 1, Figure S1). In summary, GREMLIN's automatic procedure for alignment construction may provide a more cared set of interacting proteins only when they are prokaryotic, while I-COMS procedure favors a simpler but wider applicable approach using a procedure already tested for similar purposes (22). We consider that it is a reasonable trade off between recruiting a wider set of sequences and species, automatization and performance (in terms of quality of the results) versus restricting MSA construction to only prokaryotic sequences for which there are annotated genomes with intergenic distances (Supplementary file 1, Supplementary Figure S1 and Table S1).

**Figure 1.**
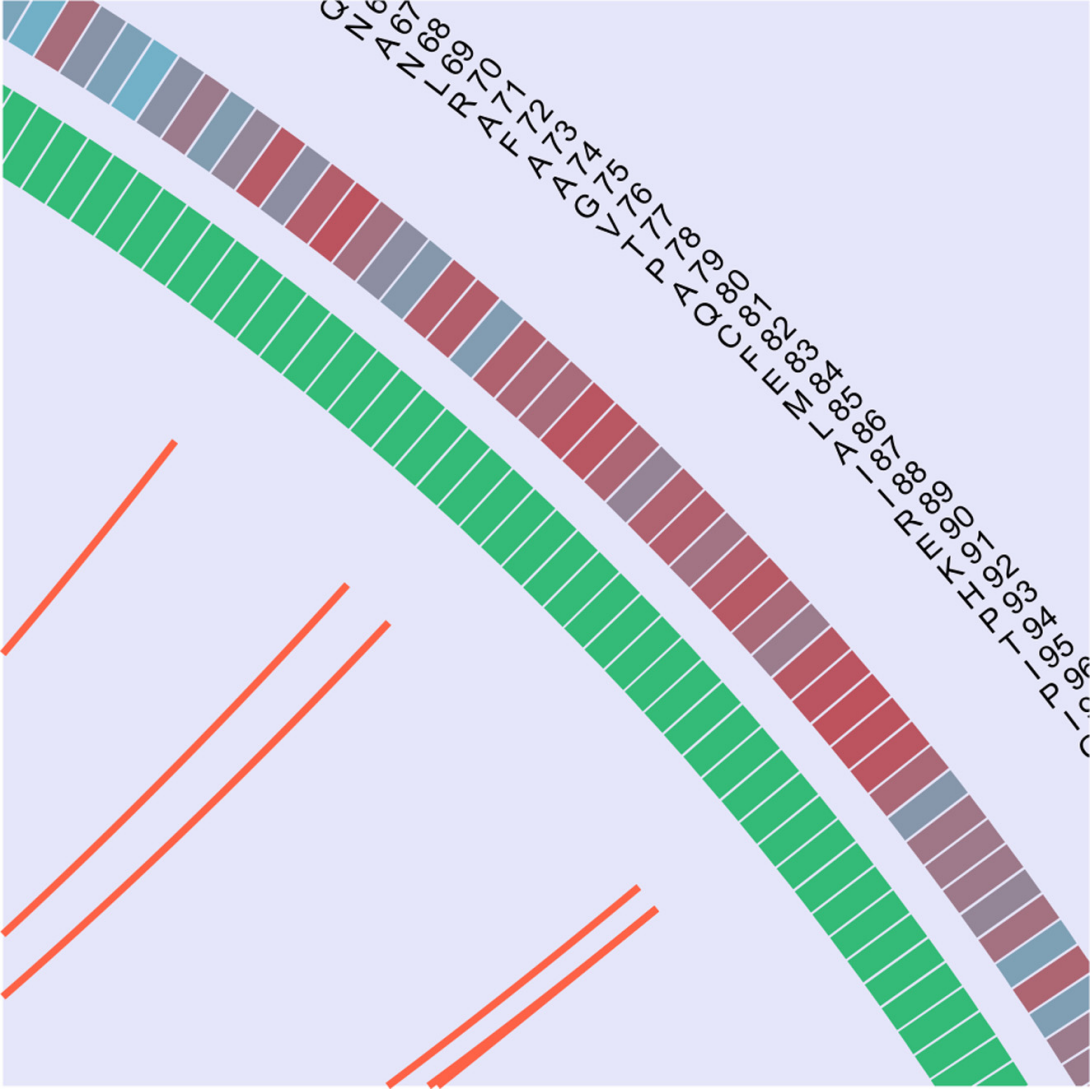
Circos representation. The outer circos's data track shows the reference sequence residue type and number. The next track (going inward) shows the conservation of that position in the MSA (from red to blue to depict higher to lower conservation respectively). The next track shows to which protein corresponds that position (colored green and violet). Finally, the inner part of the circos shows Bezier curves connecting covarying positions.

**Figure 2.**
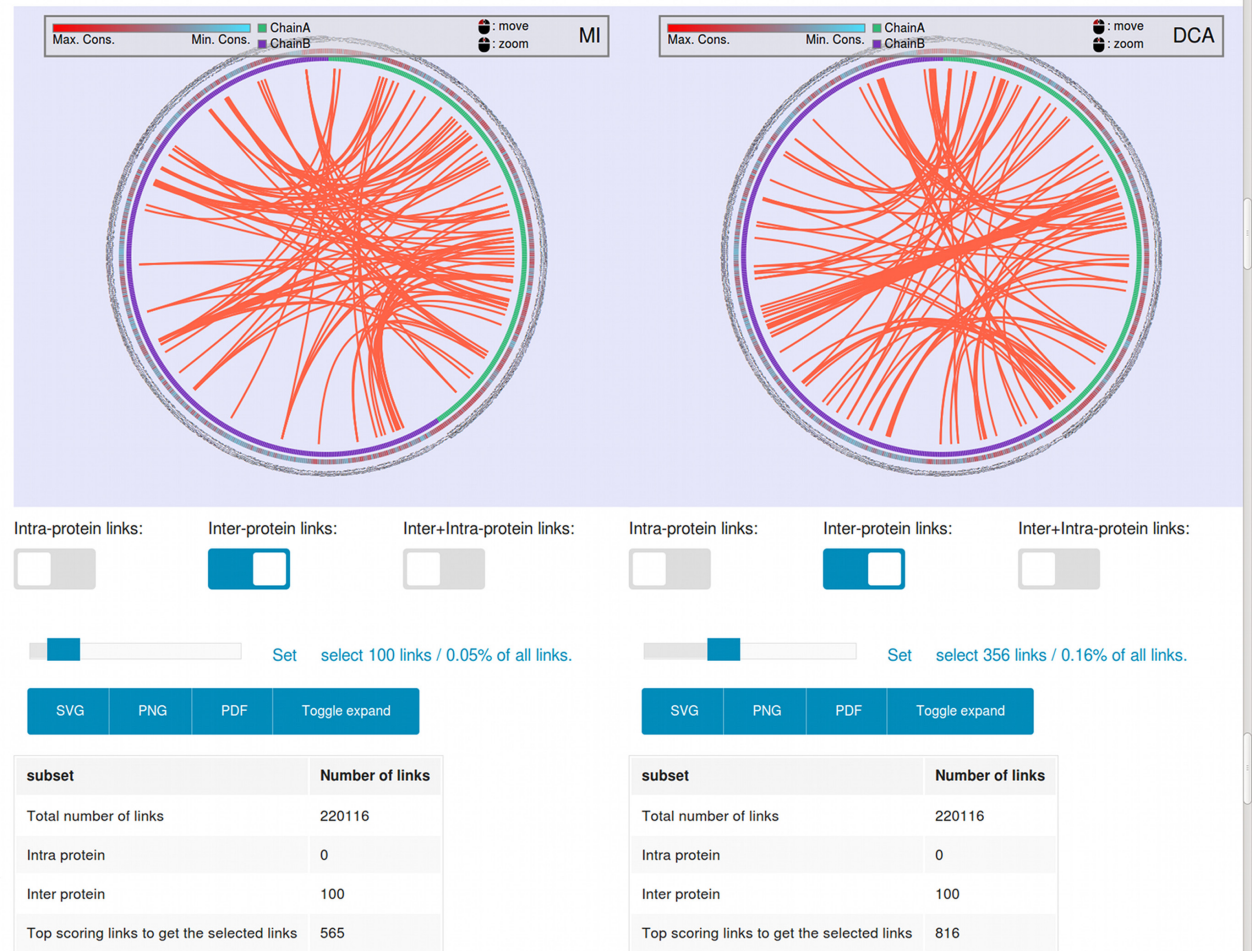
Snapshot of the single method representation. Right panel: MI method. Left panel: mfDCA method. The shown pairs are the set (selected by the user) of top scoring covarying pairs. The color of the Bezier lines represents the origin of the connected residues. If the curves are green, they both belong to the first protein, if they are violet they both belong to the second protein and if the curves are red then those covarying positions belong to different proteins.

### Software implementation

The web server was developed on Ubuntu 12.04 linux OS and is running on an Apache 2 server and PHP 5.3 as server-side scripting language. The server pipeline was written in perl 5 and interacts with scripts written in python 2.7, Java 7 and C/C++. This is mostly due to the different natures of the code used by each algorithm. The interactive graphical output in the client-side was written in Javascript. Its execution relies entirely on the web browser and was tested on the latest versions of Firefox and Chrome.

### Inputs

The MSAs can be generated by the server or loaded by the user. The input for automatic MSA construction are (i) the UniProt primary accession numbers or (ii) a PDB file (iii) the protein sequences in one letter code, of the two or more (up to six) proteins to be analyzed.

When loading each protein, the server will display the Pfam domains in order to choose whether to work with a specific domain or with the whole length protein. These sequences are used as seed to build the MSAs.

The server allows loading two or more (up to six) protein families MSA in fasta format. Those MSA should meet some requirements: they must have the same number of sequences, and sequences at the same position in each MSA must belong to the same genome or species (same taxID).

The first sequence of each MSA is assumed to be the reference sequence; all results shown are based on these sequences. In all cases MSAs were gap trimmed to remove positions with gaps in a reference sequence. In addition sequences covering <50% of the reference sequence length are removed.

The construction of these alignments may be difficult and time consuming to inexperienced users; therefore we offer a tool to build them automatically.

The user can choose which methods to run, including corrected MI, mfDCA, CCMpred and PSICOV. All methods are set with default parameters, but by opening the ‘advanced options’ menu, the parameters for each method can be customized by the user.

The server limitations are: six proteins at a time; 2000 residues are allowed as the sum of all proteins length and there is no limit on the number of protein sequences in the MSAs.

### Outputs

The server builds an MSA for each individual protein (if not provided by the user) and also a concatenated (paired) MSAs for every combination of two MSAs.

After calculation, the number of columns, the number and clusters of sequences at the selected identity value of each alignment is informed. The number of clusters is computed using Hobohm-1 algorithm (24). Note that some of the methods may require a certain number of sequences to give an accurate covariation score. All the alignments and the covariation raw data are downloadable as plain text for further analysis.

### Graphical displays

A circos representation of the covariation scores of each of the selected methods and protein pairs are displayed.

The circos representation is a rich piece of information: the outer circle shows the residue type and number of the reference sequence. The next circle shows the position conservation (as calculated in (25)) of each position colored from light blue (less conserved) to red (most conserved). The last circle shows which protein the residues belong to as a violet or green square (protein A and B, respectively). At last, the covariation scores between pair of positions are displayed as lines in the inner part of the circle (Figure 1).

When moving on top of each line, the covarying positions are displayed in the upper left corner.

If more than one method is run, two circos panels can be displayed at once. The user can load the circos for each method in the left and right panels (Figure 2). A novel feature of this type of visualization is that the user can chose to display either the ‘intra’, ‘inter’ or ‘intra + inter’ protein covariation scores by clicking in the box below the circos. Another interesting feature is that the user can control the observed number (and%) of links to be shown by a scroll bar below the representation.

Hence, the user is always in control of the visualization. As an example, the top 100 intraprotein links, the top 100 interprotein links or the top 100 intra+interprotein links could be observed. In the example in Figure 2, to observe 100 interprotein links using MI score, which are the 0.05% of the total links (220 116 total links), the first 565 top scored pairs should be inspected. Similarly, for mfDCA calculation, the 816 top scored pairs should be inspected in order to see 100 interprotein links.

This information is useful in order to take decisions about how well ranked are the interprotein covariation scores (for example, to suggest interaction) when compared to the intraprotein score of each of the single proteins. For this same reason, the server informs the score density plot of each method where the distribution of the interprotein and intraprotein scores can be observed (Supplementary Figure S3).

A foremost feature is that the overlap of the methods can be analyzed. In that section the user can visualize the overlap between any methods including between the four methods together. In the same fashion as with one method, either the intraprotein, interprotein or both types of links can be observed.

At last, a covariation matrix for each of the methods can be displayed. The square box in the miniature on top of the panel is a navigator panel, it shows which part of the matrix is visualized and permits easy navigation through the entire matrix. Pair covariation scores are shown as points in the matrix colored upon the score rank (dark red top 100 scores, orange top 500 scores and yellow top 2500 scores) (Supplementary Figure S4). The matrix is symmetric and only the upper triangular matrix is displayed.

Finally, both the circos and the matrix images can be downloaded as Portable Network Graphics, Scalable Vector Graphics or Portable Document Format files.

### Case example

The H and G chains of the *Escherichia coli* ATP synthase complex (26) is automatically loaded into the server as an illustrative example of use. We chose this example as it was shown to be a false-negative case of complex prediction with the EVcomplex score (13). The authors propose that it may be the cause of the transience of their interaction or it may reflect a lack of conservation of this interaction across the aligned proteins from different species. We analyzed the top 15 interprotein score coincidence between the four methods (MI, mfDCA, PSICOV and CCMpred) from the 90 525 total of pairs (i.e. the number of interprotein and intraprotein links with higher scores than the lowest score of the interprotein selected threshold).

**Figure 3.**
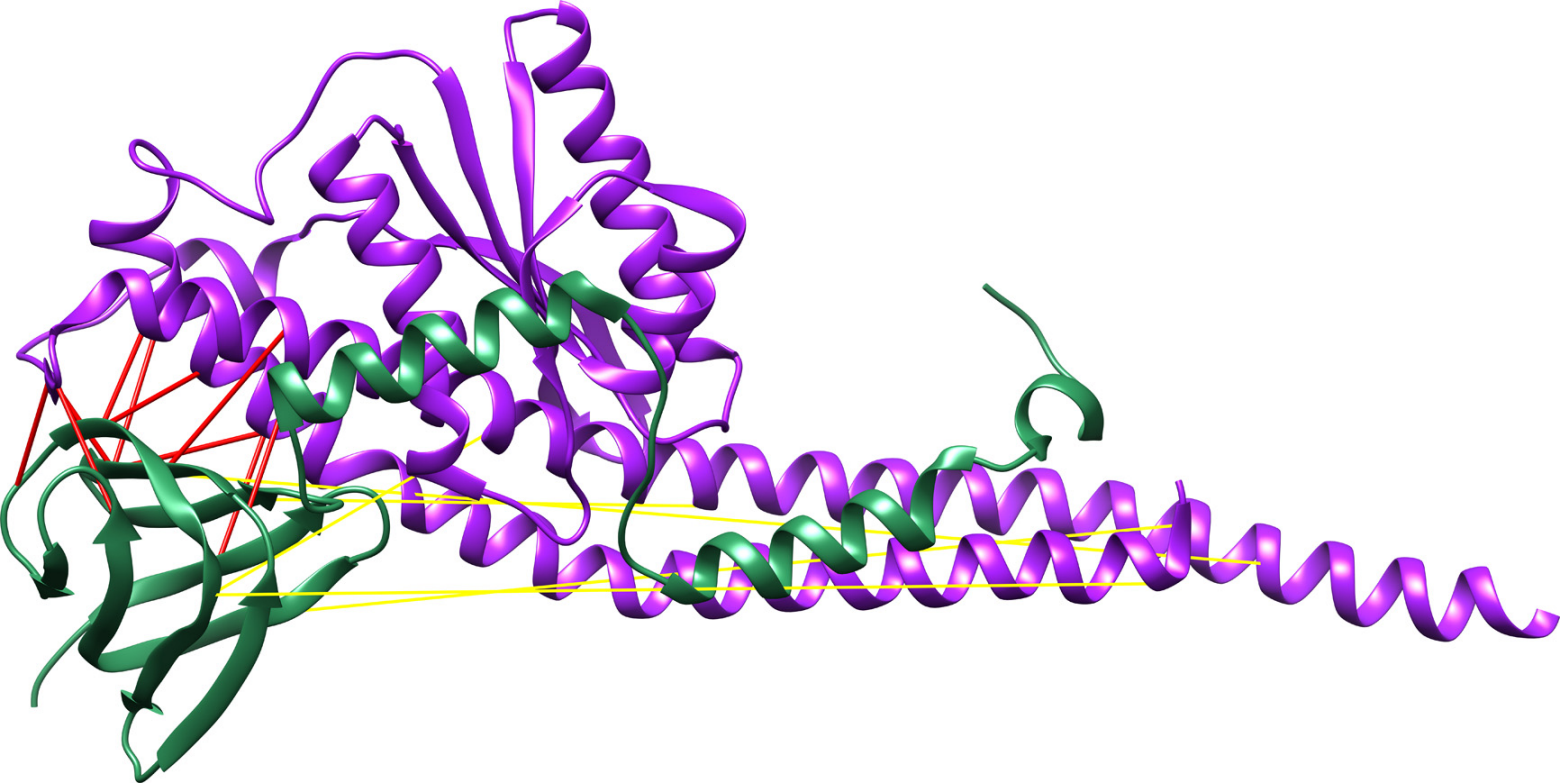
Top 15 interprotein scores overlapped between the four methods. Structure of the *E. coli* F1-ATP synthase (pdb: 3OAA), chain H (green) and G (violet). The distance of the top scoring pairs is depicted with red lines (Cα distance < 12 Å) and yellow lines (otherwise).

Surprisingly, 10 out of 15 of the scored pairs that result from overlapping the four methods are at contact distance in the protein structure (Figure 3). These 15 overlapped interprotein pairs are comprised in the top 347 pairs. In this particular case, for such number of observations the overlap of the four methods strongly benefits obtaining the truly contacting pairs between the proteins. We do not know if it holds true for other cases, to test that hypothesis a large benchmark should be assayed which is out of the scope of this paper.

We show an example of prioritization between docking models in an attempt to find a biological complex between Rab (RAB7A_HUMAN) and Ubiquitin (UBC_HUMAN) in the supplementary file. Rab proteins are small GTPases of the Ras oncoprotein family involved in the regulation of intracellular membrane traffic in mammalian cells and Ubiquitin is a regulatory protein that makes post-translational modifications that affects the proteins fate in many ways. The structure of each individual protein is known, there is evidence of interaction but no complex structural information is available (Supplementary Figure S5).

## CONCLUSIONS

Covariation analysis between proteins is an active field of work. Several computational tools have been developed in the last years. However, comparison of the results between methods is not an easy task. The web server presented here is aimed to help computing and comparing covariation between proteins using three relevant methods. To make this task easier, we focus in showing the results in a graphical and interactive way, therefore users can dynamically explore data without any additional software other than a web browser.

The server goal is to provide an environment for methods comparison. Results might be used to prioritize protein positions in a mutation analysis based on their inter and intra (if part of a complex) coevolutionary role. It might also provide a guide for docking experiments and to gain insights into protein–protein interaction when a structure of the complex is not available.

This web server generates intuitive and informative graphical representations for this type of data. For different user needs, we allow users to download raw covariation data and alignments for further analysis. Also, we provide a form to ask for new features or propose new methods, which allows any user to suggest new ways of representing results or analysis to be included in the server.

## SUPPLEMENTARY DATA

Supplementary Data are available at NAR Online.

SUPPLEMENTARY DATA
