# Supplementary material for: I-COMS: Interprotein-COrrelated Mutations Server
Source: Nucleic Acids Res. 2015 Jun 1;43(Web Server issue):W320–5. doi: 10.1093/nar/gkv572 (PMC4489276; doi:10.1093/nar/gkv572)
Supplement: SUPPLEMENTARY DATA [file supp_43_W1_W320__index.html]

I-COMS: Interprotein-COrrelated Mutations Server — I-COMS: Interprotein-COrrelated Mutations Server — SUPPLEMENTARY DATA 

# I-COMS: Interprotein-COrrelated Mutations Server

## SUPPLEMENTARY DATA

- SUPPLEMENTARY DATA
