# Supplementary material for: I-COMS: Interprotein-COrrelated Mutations Server
Source: Nucleic Acids Res. 2015 Jun 1;43(Web Server issue):W320–5. doi: 10.1093/nar/gkv572 (PMC4489276; doi:10.1093/nar/gkv572)
Supplement: SUPPLEMENTARY DATA [file supp_gkv572_nar-00612-web-b-2015-File005.pdf]

**I-COMS: Interprotein-COrrelated Mutations Server**

Javier Iserte, Franco L. Simonetti, Diego J. Zea, Elin Teppa and Cristina Marino-Buslje.

URL: http://I-COMS.leloir.org.ar/

**Supplementary file 1**

**On the paired MSA construction**

We use the same methodology as Ochoa and Pazos (Bioinformatics 2010) to build pairs of MSAs of orthologous proteins to calculate co-evolution. In this paper, the authors searched the Integr8 database of complete genomes with BLAST and retained only 1 sequence per organisms as the putative ortholog, being the others removed. From all the sequences belonging to a given organism, the one with the highest percentage identity to the query is chosen. Our server also blasts each query sequence against Uniprot-KB, and then we also retain the hit with the best e-value for any given organism as the putative ortholog. A citation to Ochoa and Pazos´s work was included in this version of the manuscript.

It is very well known that covariation results are highly dependent on the input MSA. We are aware that the proposed method to build the alignment is naive but allows to successfully determining covariation positions between proteins. Each method may perform better adjusting different alignment parameters such as number of sequences, number of positions in the MSA, sequence divergence and many other variables. However, the presented approach for building paired alignments is an opportunity for average users to be able to perform this kind of analysis. It is also a trade off between retrieve, analyze and align a large number of sequences from diverse species and the time and computational resources available. It is a simple and automatic approach, only to be used in case a curated alignment is not available.

Assessing how each method performs depending on the way each MSA was built is out of the scope of this paper. However, to test to which extent the alignments are comparable, we ran a test on a subset of Ovchinnikov (2014) dataset of complexes that have less than 650 columns in the paired alignment (15 out of 32 complexes). We built the paired MSAs using the I-COMS procedure for the same protein queries. We ran mfDCA, MI and CCMpred on both, the original MSAs (downloaded from GREMLIN page: http://openseq.org/cplx.php?mode=pdb) and the I-COMS built MSAs. The performance of each method on both types of alignments measured as the areas under the ROC curve (AUCs) for inter and inter-intra protein contacts is shown in the figure below (added as supplementary material in the manuscript). I-COMS alignments are naturally wider than GREMLIN alignments (that consider mostly bacteria), so we also tested to which extent the second are contained in the first. We compared the TaxID including in both MSAs, on average 70% of TaxIDs present in GREMLI's MSAs were also found in the MSAs generated with I-COMS methods.

Considering sequences with identical TaxID in both MSA, the retrieved protein pairs is the same 80% of the times (on average for the 15 alignments).

As shown, the differences in the alignment building procedures make the same covariation methods perform slightly different. However, we consider that it is a reasonable trade off between working on a wider set of sequences and species using an automated method, and performance. Using intergenic distances for finding orthologous is only possible in some cases and a reciprocal blast is time demanding and would be unfeasible with our computational resources.

In summary, GREMLIN procedure for automatic alignment construction may provide a more cared set of interacting proteins only when they are prokaryotic, while I-COMS procedure favors a simpler but wider applicable approach using a procedure already tested for similar purposes (1). We consider that it is a reasonable trade off between recruiting a wider set of sequences and species, automatization and performance (in terms of quality of the results) versus restricting MSA construction to only prokaryotic sequences for which there are annotated genomes with intergenic distances (Supplementary file 1, Figure S1 and table S1). The number of sequences in each alignment retrieved from GREMLIN and constructed by I-COMS is provided in Supplementary table 1.

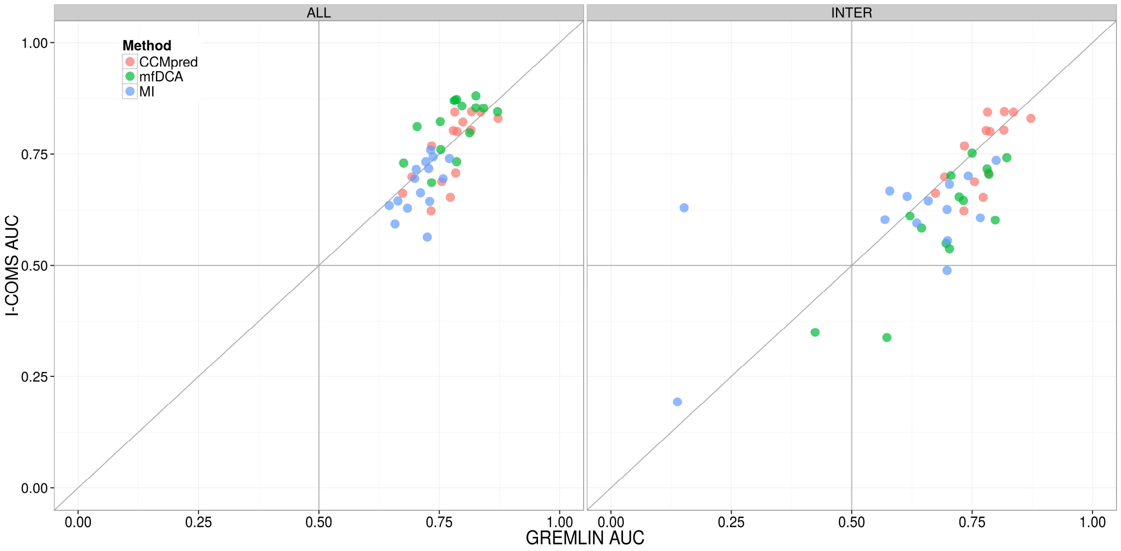


**Figure S1:** AUCs obtained with I-COMS alignments vs GREMLIN alignments for each covariation method. The y=x line is shown. If methods were to perform equally, all the points should be on this line.

**Supplementary table 1:**

| PDB code | Chains | I-COMS | GREMLIN |
|---|---|---|---|
| 3MML | A & B | 13383 | 1067 |
| 2ONK | A & C | 9277 | 4823 |
| 1TYG | B & A | 1275 | 746 |
| 3G5O | A & B | 1748 | 904 |
| 1RM6 | A & C | 8293 | 1534 |
| 1RM6 | A & B | 5228 | 1604 |
| 1RM6 | B & C | 5250 | 1481 |
| 2Y69 | A & C | 5531 | 863 |
| 2Y69 | A & B | 6835 | 1484 |
| 1I1Q | A & B | 9703 | 1204 |
| 1QOP | A & B | 17428 | 1155 |
| 1BXR | A & B | 18413 | 1004 |
| 3OAA | H & G | 17613 | 886 |
| 2NU9 | A & B | 16547 | 798 |
| 1W85 | A & B | 10816 | 1537 |
| 4HR7 | A & B | 15153 | 905 |
| 1B70 | A & B | 18980 | 1108 |
| 1EFP | A & B | 10901 | 1347 |
| 2D1P | B & C | 4172 | 216 |
| 1EP3 | A & B | 4854 | 552 |
| 3RRL | A & B | 8297 | 1330 |
| 3RPF | A & C | 467 | 753 |
| 3IP4 | A & B | 15166 | 782 |
| 3IP4 | A & C | 12645 | 879 |
| 3IP4 | B & C | 12629 | 689 |
| 2Y69 | B & C | 5183 | 1408 |
| 2WDQ | C & D | 5844 | 221 |
| 3PNL | A & B | 10564 | 902 |
| 2VPZ | A & B | 4348 | 676 |
| 1ZUN | A & B | 9736 | 649 |
| 1IXR | A & C | 19123 | 829 |
| 3A0R | A & B | 125 | 6732 |

**Supplementary table1**: Number of sequences in the MSAs generated by GREMLIN and I-COMS. PDB codes and chains correspond to the published GREMLIN data set. For building I-COMS MSAs, protein sequences were extracted from each PDB and used as seeds for I-COMS workflow.

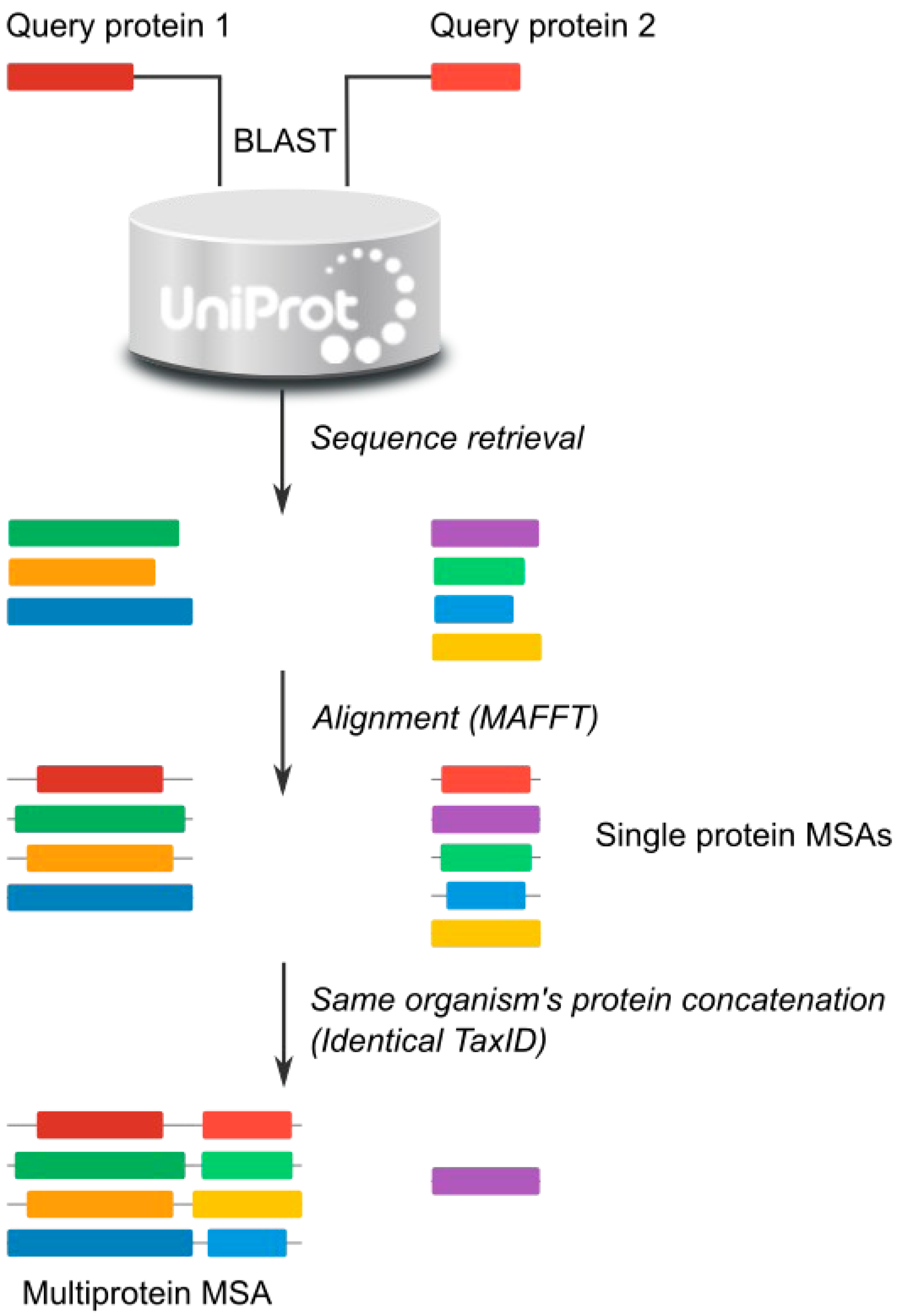


**Figure S2:** Schematic view of the workflow from the input sequences to construction of the multiprotein MSA. The retrieved sequences are aligned building single protein MSAs, then a multiprotein MSA is constructed by pairing sequences with identical TaxID. Unpaired sequences are discarded (represented in violet).

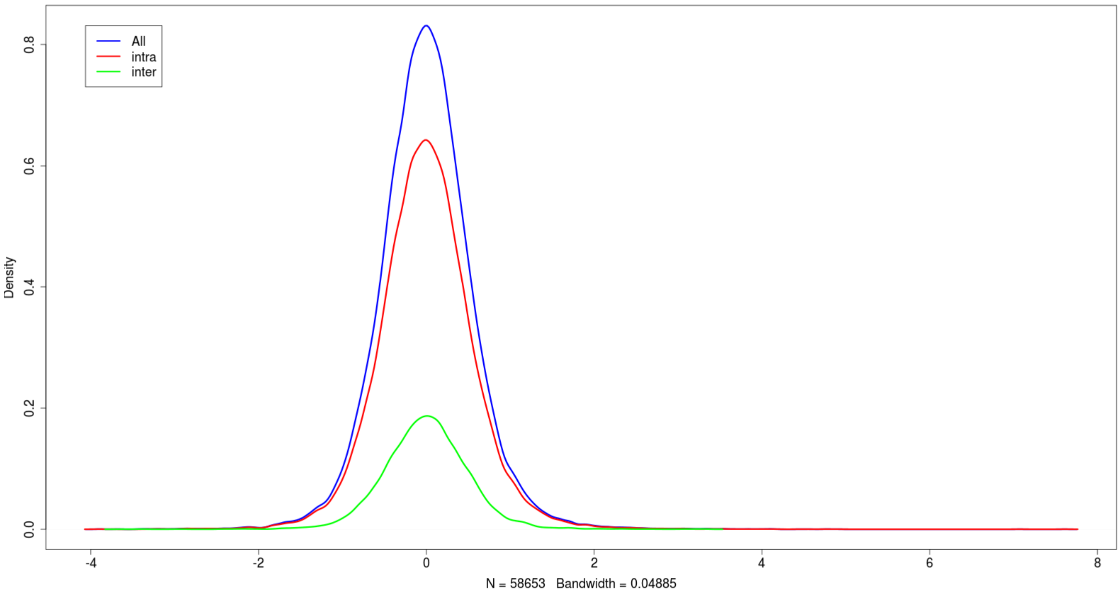


**Figure S3:** Distribution of covariation scores as a probability density function. Colored blue are all covariation scores calculated for a protein pair. Red and green depicts the intra and inter protein covariation scores respectively.

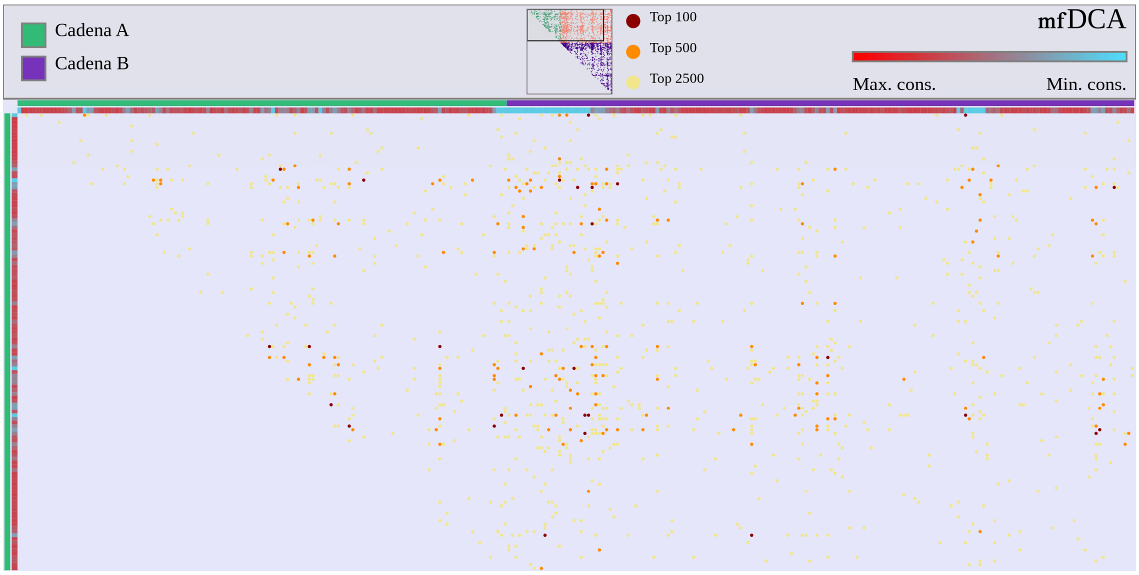


**Figure S4:** Matrix representation of the covariation scores for a pair of proteins. The square box in the miniature on top of the panel is a navigator panel, it shows which part of the matrix is visualized and permits easy navigation through the entire matrix. Green, violet and red are used in the navigator to show areas of intra (green and violet) and inter protein scores. Pair covariation scores are shown as points in the matrix colored upon the score rank (dark red top 100 scores, orange top 500 scores and yellow top 2500 scores). Conservation of each position in the input MSA is also shown as red/blue bars at the top and left side of the matrix. The matrix is an interactive element that can be zoomed in/out and panned by the user.

**Example of prioritization between docking models in an attempt to find a biological complex:**

We take as example a Rab (RAB7A_HUMAN) Ubiquitin (UBC_HUMAN) protein interaction present in the BioGRID (2) and STRING (3) databases. The structure of both proteins is known but there is no structure information for the complex. Rab proteins are small GTPases of the Ras oncoprotein family involved in the regulation of intracellular membrane traffic in mammalian cells. Ubiquitin is a small regulatory protein that has been found ubiquitously in almost all tissues of eukaryotic organisms. Ubiquitination is a post-translational modification where ubiquitin is attached to a substrate protein and this affects the proteins fate in many ways**.** We performed a docking study between both proteins with Zdock server (4). In figure S5A is shown that there are very different possible orientations between the proteins in the top 10 scored complexes (supplementary figure 5A). Taken into account the top 10 overlapped covariation scored pairs, we prioritise the 5 solutions shown in figure 5B, and hopefully, the actual biological complex will be close to the ones shown in figure 5C and D. However, further evidence is needed to validate this hypothesis.

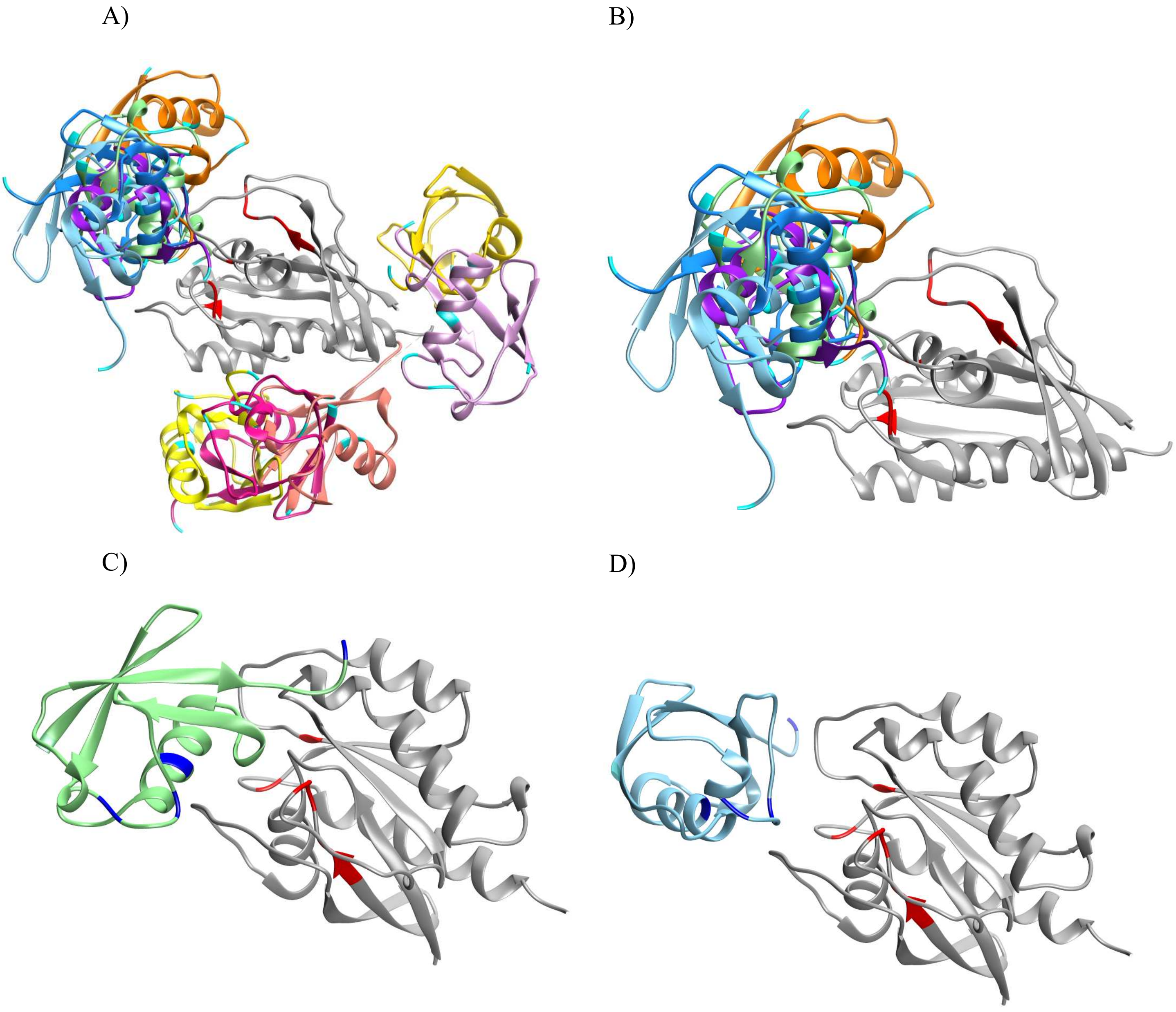

**Figure S5:** Ribbon representation of the putative RAB7A_HUMAN-UBC_HUMAN complex. RAB7A_HUMAN is coloured gray (pdb code: 3LAW). UBC_HUMAN (pdb code: 1D3Z) takes different colors for different solutions (panels A and B). Top 10 predicted interacting pairs are coloured red in RAB7A and cyan in UBC (panels A and B). For a better visualization, UBC residues were coloured blue in panels C and D.

## References:


1. Ochoa, D. and Pazos, F. (2010) Studying the co-evolution of protein families with the Mirrortree web server. *Bioinformatics (Oxford, England)*, **26**, 1370-1371.
2. Chatr-Aryamontri, A., Breitkreutz, B.J., Oughtred, R., Boucher, L., Heinicke, S., Chen, D., Stark, C., Breitkreutz, A., Kolas, N., O'Donnell, L. *et al.* (2015) The BioGRID interaction database: 2015 update. *Nucleic Acids Res*, **43**, D470-478.
3. Szklarczyk, D., Franceschini, A., Wyder, S., Forslund, K., Heller, D., Huerta-Cepas, J., Simonovic, M., Roth, A., Santos, A., Tsafou, K.P. *et al.* (2015) STRING v10: protein-protein interaction networks, integrated over the tree of life. *Nucleic Acids Res*, **43**, D447-452.
4. Pierce, B.G., Wiehe, K., Hwang, H., Kim, B.H., Vreven, T. and Weng, Z. (2014) ZDOCK server: interactive docking prediction of protein-protein complexes and symmetric multimers. *Bioinformatics (Oxford, England)*, **30**, 1771-1773.
